# Cognitive deficits associated with impaired awareness of hypoglycaemia in type 1 diabetes

**DOI:** 10.1007/s00125-017-4233-3

**Published:** 2017-03-09

**Authors:** Tor I. Hansen, Sandra E. Olsen, Elise C. D. Haferstrom, Trond Sand, Brian M. Frier, Asta K. Håberg, Marit R. Bjørgaas

**Affiliations:** 1 Department of Neuromedicine and Movement Science, Faculty of Medicine and Health Sciences, NTNU – Norwegian University of Science and Technology, Trondheim, Norway; 2 Department of Radiology, St Olavs Hospital, Trondheim University Hospital, Trondheim, Norway; 3 Department of Cancer Research and Molecular Medicine, Faculty of Medicine and Health Sciences, NTNU – Norwegian University of Science and Technology, PO Box 8905, N-7491 Trondheim, Norway; 4 Department of Neurology and Clinical Neurophysiology, St Olavs Hospital, Trondheim University Hospital Trondheim, Trondheim, Norway; 5 British Heart Foundation Centre for Cardiovascular Science, The Queen’s Medical Research Institute, University of Edinburgh, Edinburgh, UK; 6 Department of Endocrinology, St Olavs Hospital, Trondheim University Hospital, Trondheim, Norway

**Keywords:** Cognitive function, Hypoglycaemia, Impaired awareness of hypoglycaemia, Memory, Pattern separation, Type 1 diabetes

## Abstract

**Aims/hypothesis:**

The aim of this study was to compare cognitive function in adults with type 1 diabetes who have impaired awareness of hypoglycaemia with those who have normal awareness of hypoglycaemia. A putative association was sought between cognitive test scores and a history of severe hypoglycaemia.

**Methods:**

A total of 68 adults with type 1 diabetes were included: 33 had impaired and 35 had normal awareness of hypoglycaemia, as confirmed by formal testing. The groups were matched for age, sex and diabetes duration. Cognitive tests of verbal memory, object-location memory, pattern separation, executive function, working memory and processing speed were administered.

**Results:**

Participants with impaired awareness of hypoglycaemia scored significantly lower on the verbal and object-location memory tests and on the pattern separation test (Cohen’s *d* −0.86 to −0.55 [95% CI −1.39, −0.05]). Participants with impaired awareness of hypoglycaemia had reduced planning ability task scores, although the difference was not statistically significant (Cohen’s *d* 0.57 [95% CI 0, 1.14]). Frequency of exposure to severe hypoglycaemia correlated with the number of cognitive tests that had not been performed according to instructions.

**Conclusions/interpretation:**

Impaired awareness of hypoglycaemia was associated with diminished learning, memory and pattern separation. These cognitive tasks all depend on the hippocampus, which is vulnerable to neuroglycopenia. The findings suggest that hypoglycaemia contributes to the observed correlation between impaired awareness of hypoglycaemia and impaired cognition.

**Electronic supplementary material:**

The online version of this article (doi:10.1007/s00125-017-4233-3) contains peer-reviewed but unedited supplementary material, which is available to authorised users.

## Introduction

A diminished ability to perceive the onset of hypoglycaemia occurs in 17–25% of people with type 1 diabetes [1, 2]. Impaired awareness of hypoglycaemia (IAH) is a major risk factor for severe hypoglycaemia (SH), defined as an event requiring external assistance, and increases the risk of SH sixfold [1, 3]. Cognitive decline may be a complication of longstanding type 1 diabetes [4], and several cognitive domains seem to be affected [4–7]. Cognitive dysfunction may therefore contribute to suboptimal diabetes management, including the avoidance and treatment of hypoglycaemia. In support of this hypothesis, some adults with IAH do not modify their behaviour to prevent or avoid hypoglycaemia [3], and fail to adhere to recommended therapeutic measures [8].

Recurrent exposure to hypoglycaemia is strongly implicated in the pathogenesis of IAH [9]. A putative association between IAH and impaired cognitive function may therefore exist, since both could be the consequence of recurrent SH, the frequency of which is promoted by IAH [1, 3]. Alternatively, for people with type 1 diabetes who have premorbid cognitive dysfunction, self-management may be suboptimal, thereby increasing the risk of IAH. Furthermore, the development of IAH and impaired cognitive function may have a common predisposing factor. If IAH is associated with premorbid cognitive dysfunction, impairment should involve several cognitive domains. Alternatively, if cognitive impairment in people with IAH is caused by recurrent SH, then we would expect cerebral functions dependent on brain regions that are vulnerable to hypoglycaemia to be diminished.

A causal association between recurrent SH and cognitive impairment in adults with type 1 diabetes is unproven. Anecdotal reports have described memory loss following SH [10–12], and cross-sectional studies have demonstrated impairment of several cognitive domains in adults with a history of SH [13–16]. However, the Epidemiology of Diabetes Interventions and Complications (EDIC) study (the follow-up to the DCCT) and a smaller Swedish prospective study both found that recurrent SH had little or no adverse effect on cognition in adults with type 1 diabetes [17, 18], a conclusion supported by a meta-analysis [5]. A more recent meta-analysis concluded that reduced memory and executive function are associated with SH [7], which people with IAH experience at a much higher frequency than was recorded in the DCCT/EDIC study. SH may cause localised neuronal death within the hippocampus and cerebral cortex, and in white matter, as demonstrated histologically and in vivo with MRI after SH in animals and humans [11, 19–21]. It is therefore plausible that recurrent SH could compromise cognitive functions that are dependent on brain regions particularly sensitive to neuroglycopenia.

Three previous studies in the early 1990s found a possible association between IAH and cognitive impairments, including memory impairment, selective attention and a trend towards reduced intelligence quotient. These investigators hypothesised that the impairments resulted from frequent exposure to SH, as experienced by people with IAH [22–24]. However, putative associations between IAH, recurrent hypoglycaemia and cognitive dysfunction have remained unresolved.

The aim of the present study was to compare cognitive function in people with type 1 diabetes who had established IAH, with those in whom hypoglycaemia awareness remained intact. For this purpose, tests of verbal memory, object-location memory, pattern separation, working memory, information processing speed and executive function, including planning, were applied. Optimal cognitive function depends on interaction within networks of brain regions. For learning, memory, and pattern separation abilities, the most central structure for normal functioning is the hippocampus [25, 26], while executive functions, working memory and information processing speed depend on frontal and parietal cortices and their connectivity [27, 28]. The intention was to test cognitive abilities that depend on brain regions susceptible to damage during hypoglycaemia [11, 21] and cognitive abilities that are recognised to be impaired in patients with type 1 diabetes [4, 5]. Finally, because many people with IAH do not modify their behaviour to avoid SH [3], exemplified by some failing to measure their blood glucose in relation to driving [29, 30], executive functions that include planning ability [31] and pattern separation, which can affect a person’s ability to identify a hypoglycaemic episode, were assessed. A secondary aim was to assess whether cognitive function in participants with IAH is related to their historical SH burden.

## Methods

### Participants

Adults with type 1 diabetes with IAH (Gold score ≥4) and with normal hypoglycaemia awareness (Gold score 1–2) [3] were recruited from a cross-sectional survey of the outpatient population with type 1 diabetes attending St Olavs Hospital, Trondheim, Norway [2]. In that survey, questionnaires were posted to 636 adults with type 1 diabetes and returned by 70%, with 440 questionnaires suitable for analysis. From these, 74 people with IAH were identified (17%). Autonomic dysfunction and peripheral neuropathy has previously been investigated in this patient sample [32]. The present study excluded people with IAH aged >65 years (*n* = 7), people who used medication that could influence test results (*n* = 5) and those with severe comorbidity such as previous head injury, psychiatric, neurological or other systemic disease, or reduced vision or hearing (*n* = 6).

In total, 56 people with IAH were eligible; of these, 33 agreed to participate. From those people with normal hypoglycaemia awareness (NAH) in the survey, individuals of the same sex and of similar age and diabetes duration (±5 years) were selected at random. In total, 59 people with NAH were identified as possible participants. Of these, 16 were not eligible (age >65 years, *n* = 4; neurological comorbidity, n = 8; other severe comorbidity, *n* = 4), and eight declined to participate. The NAH group therefore comprised 35 participants

Diabetes duration was confirmed from hospital records. On the day of testing, participants documented their insulin regimen, frequency of self-monitoring of blood glucose (SMBG), and current medication in a questionnaire. They also reported the occurrence of SH experienced in the preceding year (no episode, 1–2 episodes or ≥3 episodes) and from diagnosis (no episode, 1–2 episodes, 3–5 episodes or ≥6 episodes). Educational history was categorised into: level 1, grades 1–10, primary/lower secondary school; level 2, upper secondary school; level 3, ≤4 years at university/college; and level 4, >4 years of tertiary education. Blood and urine samples were obtained for the measurement of HbA_1c_ and albumin/creatinine ratio, respectively, and BP was measured. To corroborate the classification of hypoglycaemia awareness, participants’ Gold scores were re-measured [3] and Clarke scores were completed [33]. Data from routine ophthalmological assessment were obtained from case records.

The study was approved by the regional medical ethics committee (2012/439). All participants gave written informed consent.

### Preparations and precautions

As antecedent hypoglycaemia may influence cognitive test performance [34], participants were requested to apply less strict glycaemic targets for 24 h before cognitive testing. Furthermore, they were requested to abstain from drinking alcohol, to perform frequent SMBG to avoid plasma glucose values of <4.0 mmol/l and not to exercise for 24 h before testing. Tests were postponed if SH had occurred within the previous week.

### Cognitive tests

Six cognitive tests were applied via the self-administered web-based neuropsychological test platform, Memoro (Trondheim fMRI group, NTNU, Norway) [35, 36]. The cognitive tests assessed verbal and object-location memory, working memory, planning abilities, and coding. In addition, the ability to learn and recall distinct non-overlapping representations of highly similar everyday objects, namely pattern separation [25], was assessed. See electronic supplementary material (ESM) Methods for test details. Fifty-nine participants performed the tests in a quiet room at St Olav’s Hospital. The remaining nine participants (eight participants with IAH and one participant with NAH) performed the tests at home because of long travelling distances or unavoidable work commitments. Memoro test scores have been shown not to differ when tests are performed at home compared with a controlled laboratory setting [33]; this finding was confirmed in the current dataset (lowest *p* value: Mann–Whitney *U* tests, exact significance *p* = 0.106). Participants with type 1 diabetes performed SMBG before and after testing, and plasma glucose had to be ≥4.5 mmol/l before testing could commence. Participants followed standardised aural and written instructions including pre-trial tests provided by the test platform. A research assistant was available for technical support in a nearby room (for in-hospital testing) or by telephone (for home testing). Participants also completed the Memoro short computer questionnaire, which gives a computer familiarity score [35, 36].

For illustrative purposes only, scores on each test for the two groups are displayed relative to scores from the Memoro control population (i.e. non-diabetic) database (*n* = 197). Statistical comparisons were, however, limited to IAH–NAH between-group differences, in line with the study aims.

### Blinding

TIH and AKH were blinded as to whether participants had diabetes and to their hypoglycaemia awareness status during data collection and analysis.

### Statistical analyses

#### Demographic and type 1 diabetes related data

Differences between the IAH and NAH groups were investigated using independent *t* tests for normally distributed data and Mann–Whitney *U* tests for non-normally distributed data. *χ* ^2^ or Fisher’s exact tests were performed on cross-tabular data, as appropriate.

#### Cognitive test scores

**Fig. 1:**
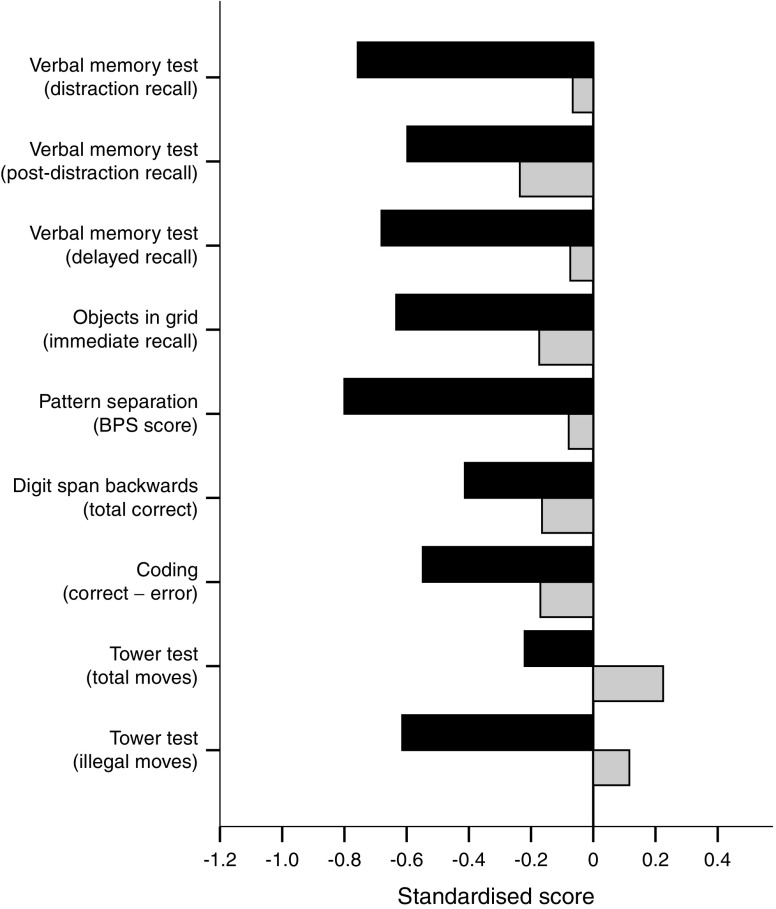
Scores for the nine cognitive measures were standardised against Memoro test norms for a non-diabetic population. BPS, behavioural pattern separation. Black bars, IAH; grey bars, NAH

All raw scores were transferred from the Memoro database to an IBM SPSS Statistics software (version 22.0, Chicago, IL, USA) data file. The Tower test illegal moves variable was log_10_ transformed to approximate a normal distribution. Test scores clearly demonstrating that a participant had misinterpreted the instructions were excluded on a case-by-case basis (for example, if a participant indicated at the start of the Pattern separation test that novel stimuli had been presented previously). For Fig. 1, the IAH and NAH raw scores were standardised against the Memoro test norms. Standardised scores for the Tower test were inverted such that a low value represents poorer performance.

Independent *t* tests were used to investigate statistical differences between the IAH and NAH groups, and Cohen’s *d* with 95% CI was calculated to estimate the effect size. Repeated measures ANOVA was used to investigate whether a difference in performance was present throughout all trials in the verbal memory test, and whether an interaction existed between groups and trials. Spearman’s rank correlation analyses were performed to investigate the association between test scores and the number of SH episodes for all participants with diabetes, and separately for the IAH and NAH groups. Linear regression models were used to investigate the relationship between test performance and plasma glucose level.

#### Statistical significance and data presentation

For all statistical tests, the threshold for statistical significance was set at *p* ≤ 0.05, two tailed. Results from normally distributed data are given as mean ± SD, and from non-normally distributed data as median and interquartile range (IQR) or frequency and percentage.

### Supplementary analyses

In the present study, awareness status had changed in five participants between the cross-sectional study of 2011 and this study. Supplementary analyses were therefore performed after excluding these participants. See ESM Table 1 and ESM Fig. 1.

## Results

### Participants

The study included 68 participants with type 1 diabetes comprising two groups: 33 with IAH (19 men) and 35 with NAH (21 men). As shown in Table 1, the two groups were of similar age, diabetes duration, and had similar BP, HbA_1c_ level, insulin regimen, prevalence of microvascular complications, educational level and computer familiarity.

**Table 1:** Characteristics of the IAH and NAH groups

Characteristic	IAH (*n* = 33)	NAH (*n* = 35)
Age, years	47 ± 10	47 ± 10
Sex (men: women), %	58:42	60:40
Age of diabetes onset, median (IQR)	16 (11–26)	13 (10–24)
Diabetes duration, years	30.1 ± 9.6	30.2 ± 10.4
Education, median (IQR)	2 (1–4)	2 (1–4)
Computer familiarity score^a^	28.0 ± 4.2	28.7 ± 5.0
Plasma glucose level, mmol/l		
Pre-testing	10.4 ± 2.9	10.3 ± 3.9
Post-testing	10.0 ± 3.2	10.0 ± 4.0
BP, mmHg		
Systolic	120.8 ± 15.9	120.0 ± 13.7
Diastolic	73.2 ± 7.8	73.5 ± 7.5
HbA_1c_ level, %	7.9 ± 1.6	8.0 ± 1.1
HbA_1c_ level, mmol/mol	63.0 ± 17.8	64.0 ± 12.1
Participants performing SMBG ≥4 times per day, *n* (%)	17 (51.5)	17 (48.6)
Insulin regimen, *n* (%)		
Long + rapid acting analogue	18 (54.5)	16 (45.7)
NPH insulin + rapid acting analogue	7 (21.2)	7 (20.0)
CSII with rapid acting analogue	8 (24.2)	11 (31.4)
Biphasic insulin	0	1 (2.9)
Retinopathy, *n* (%)		
None	14 (42.4)	16 (45.7)
Non-proliferative	14 (42.4)	14 (40.0)
Mild/moderate proliferative	5 (15.1)	5 (14.3)
Visual acuity^b^ in best eye, *n* (%)		
≥1.0	28 (84.8)	29 (82.9)
<1.0 but ≥0.70	4 (12.1)	6 (17.1)
<0.70 but ≥0.50	1 (3.0)	0 (0)
Urinary albumin-to-creatinine ratio^c^		
Median (IQR), mg/mmol	0.9 (0.55–2.0)	0.8 (0.5–2.2)
<3 mg/mmol, *n* (%)	25 (86.2)	27 (79.4)
≥3 mg/mmol, *n* (%)	4 (13.8)	7 (20.6)
SH episodes, *n* (%)		
Since diagnosis		
None	6 (18.2)	7 (20.0)
1–2	1 (3.0)	6 (17.1)
3–5	6 (18.2)	5 (14.3)
≥6	20 (60.6)	17 (48.6)
In preceding year		
None	20 (60.6)	28 (80.0)
1–2	9 (27.3)	6 (17.1)
≥3	4 (12.1)	1 (2.9)
Asymptomatic hypoglycaemia^d^ during preceding month, *n* (%)^e^		
Never	9 (27.3)	24 (68.6)
1–3 times	5 (15.2)	8 (22.9)
Once/week	2 (6.1)	1 (2.9)
≥ Twice/week	17 (51.5)	2 (5.7)

Data are mean ± SD, unless otherwise stated

^a^Self-assessment of computer familiarity, range 0–35

^b^A score of 1 = 6/6

^c^Data from 29 participants with IAH and 34 participants with NAH

^d^Plasma glucose level of <3.9 mmol/l without symptoms

^e^Fisher’s exact test = 20.558, *p* < 0.001

CSII, continuous subcutaneous insulin infusion

No differences were observed in age, diabetes duration, sex distribution, HbA_1c_ level or frequency of SH during the preceding year between the 68 participants and the 31 candidates (23 with IAH and eight with NAH) who declined participation (see ESM Table 2).

Participants with IAH recorded more episodes of asymptomatic hypoglycaemia (plasma glucose <3.9 mmol/l) during the month preceding the study (Fisher’s exact test = 20.558, *p* < 0.001; Table 1) compared with participants with NAH. In addition, participants with IAH reported a higher number of SH episodes during the preceding year, although this was not statistically significant (*χ* ^2^_1,68_ = 3.077, *p* = 0.079). None of the participants had experienced SH within 60 days of the start of the study.

No participant developed hypoglycaemia during cognitive testing, which took approximately 52 min. In two participants with NAH, plasma glucose readings were 4.3 mmol/l and 4.2 mmol/l, respectively, after performing the tests. Data from these participants were not excluded since cognitive dysfunction does not commence until the plasma glucose level approaches 3.2 mmol/l in people with type 1 diabetes [37]; this value is even lower in people with IAH [34]. The mean plasma glucose levels before and after the cognitive tests were similar in both groups (Table 1).

### Cognitive test results

The raw scores from different cognitive tests and statistical comparisons between groups are presented in Table 2. Some participants failed to perform the tests as instructed (these were denoted invalid tests); therefore, only data for participants with valid scores are shown.

**Table 2:** Results of cognitive tests and comparisons of the IAH and NAH groups

Cognitive test	IAH group		NAH group		*p*	Cohen’s *d* (95% CI)
	*n* ^a^	Mean ± SD	*n* ^a^	Mean ± SD		
Verbal memory test^b^						
Distraction recall	32	6.4 ± 2.3	35	8.3 ± 3.1	0.007	−0.70 (−1.18, −0.2)
Post-distraction recall	32	12.3 ± 2.6	35	13.2 ± 2.6	0.179	−0.34 (−0.83, 0.14)
Delayed recall	32	12.4 ± 2.2	32	13.7 ± 2.1	0.018	−0.62 (−1.11, −0.10)
Objects in grid^b^	30	7.9 ± 3.1	34	9.9 ± 4.7	0.043	−0.55 (−1.05, −0.05)
Pattern separation^b^	28	0.19 ± 0.20	33	0.35 ± 0.17	0.001	−0.86 (−1.39, −0.34)
Digit span backwards^b^	29	9.2 ± 2.7	33	9.9 ± 2.8	0.328	−0.25 (−0.76, 0.25)
Coding^b^	27	29.2 ± 8.1	34	34.4 ± 11.6	0.051	−0.52 (−1.02, 0.01)
Tower test^c^						
Total moves	24	57.5 ± 9.4	25	54.0 ± 6.6	0.142	0.43 (−0.13, 1.0)
Log illegal moves	24	0.46 ± 0.29	25	0.29 ± 0.31	0.057	0.57 (0, 1.14)

^a^Number of valid responses

^b^Higher scores indicate better performance

^c^Higher scores indicate poorer performance

**Fig. 2:**
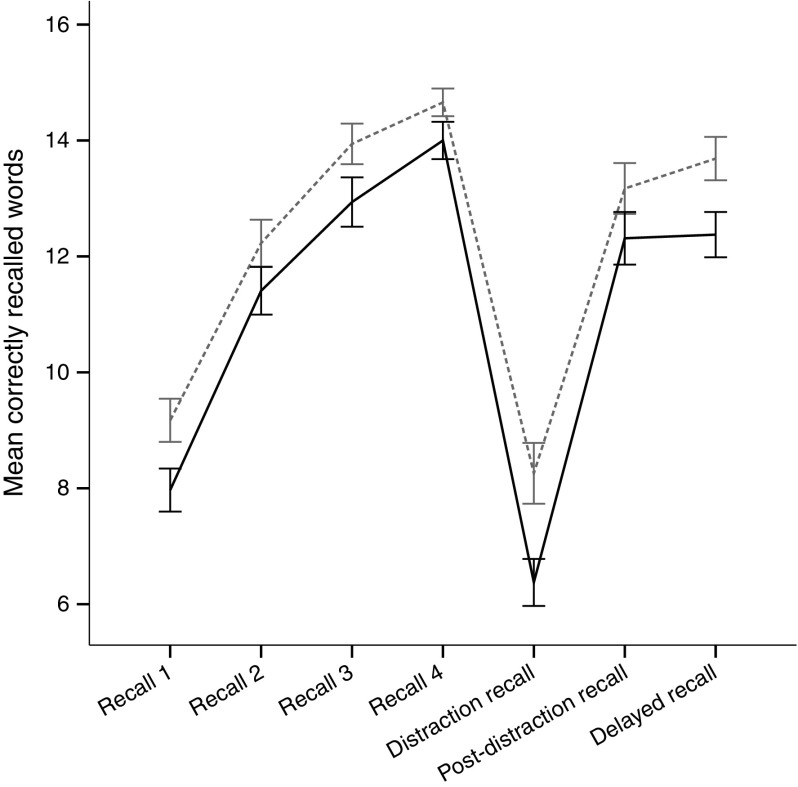
Mean (±SD) performance throughout the Verbal memory test. The between-group difference is significant (repeated measures ANOVA *p* = 0.01 with a non-significant interaction). Black solid line, IAH; grey dashed line, NAH

The scores in participants with IAH were significantly lower compared with those of participants with NAH on the Verbal memory distraction recall and delayed recall, Objects in grid and Pattern separation tests (Table 2). The verbal memory learning curves demonstrate that participants with IAH generally had poorer recall than participants with NAH (Fig. 2). A significant group effect was observed across the trials (*F*_1,60_ = 7.123, *p* = 0.010), but no interaction was found between group and trials. No differences were found between participants with IAH and NAH on the Digit span backwards, Verbal memory test post-distraction recall or Tower test total moves scores. Reduced performance in participants with IAH was observed in the Tower test illegal moves and Coding tests, although the difference was not significant (Table 2).

Plasma glucose levels after testing had a significant (*r* = 0.405, *p* = 0.02) correlation with Digit span backwards scores in the NAH group, i.e. results in this test were better in participants with higher glucose levels, but plasma glucose before or after testing did not correlate with performance on any other test in either group. Adding plasma glucose level as a covariate when comparing Digit span backwards scores for the IAH and NAH groups did not alter the results. Six participants (two with IAH, four with NAH) had plasma glucose levels of >15 mmol/l before the tests. Re-analysis excluding these participants did not affect between-group differences for any test.

Figure 2 shows a comparison of the performance of the IAH and NAH groups standardised against Memoro test norms for a non-diabetic population. In general, the performance of the IAH group was poorer compared with the NAH group across all tests. Furthermore, scores in the IAH group deviated most from the norms.

No significant correlations were demonstrated between scores for the different cognitive tests and the approximate number of SH episodes (since diagnosis or during the preceding year). However, in the IAH group, a significant correlation was found between the number of invalid tests and the number of SH episodes since diagnosis (Spearman’s ρ = 0.57, *p* = 0.026).

When the five participants whose hypoglycaemia awareness status had changed were excluded from the statistical analysis, the between-group differences for cognitive tests became more prominent (see ESM Results and ESM Fig. 1). Furthermore, a significant between-group difference for Tower test illegal moves scores emerged (*p* = 0.047, Cohen’s *d* 0.61 [95% CI 0.02, 1.19]), i.e. the performance of participants with IAH was significantly worse compared with participants with NAH; this finding had been a non-significant trend in the analysis using the original IAH cohort.

## Discussion

By employing an extensive cognitive test battery and validated methods to assess hypoglycaemia awareness in well-matched participants with type 1 diabetes, the present study demonstrated that adults with type 1 diabetes who have IAH have modestly impaired cognitive performance compared with people with NAH, thus adding further evidence to previous reports on this topic [22–24].

The IAH group exhibited significant impairment in pattern separation abilities in comparison with the NAH group, as well as on supplementary analyses of planning function (the Tower test illegal moves). Pattern separation is critical for accurate memory: decreased pattern separation ability contributes to interference among memories and convergence of similar episodes into a generalised representation rather than distinct memories [25]. It is possible that people with IAH have a diminished ability to distinguish cues that are specifically associated with hypoglycaemia and hence are unable to take appropriate action to avoid SH. Executive function measured with the Tower test in the present study assesses planning ability and, as such, a person’s capacity to adjust behaviour to current and future demands and goals [31]. The present results suggest that planning ability may be restricted in people with IAH and might underlie the observation that many people with IAH do not modify their behaviour to prevent hypoglycaemia [3] or adhere to prescribed therapy [8].

In the IAH group, significant impairments were observed in the learning, memory and pattern separation tests, all of which rely on the integrity of the hippocampus, a brain structure vulnerable to neuroglycopenic injury [11, 19, 20]. In people with type 1 diabetes, learning and memory seem to be largely unaffected [5, 17], although two studies have shown memory impairment in people with recurrent SH [24, 38]. In the present study, participants with IAH exhibited both learning difficulties and impaired delayed recall in the Verbal memory test. An IAH-specific learning deficit was also evident in the Objects in grid test, which is an object-location memory and one-trial learning test. Hence, the impairment in memory and learning in those with IAH was generalised, pertaining both to words heard and objects seen. The difference between the IAH and NAH groups in the Verbal memory test is similar to the difference observed after 7 years of ageing in a middle-aged non-diabetic population [39]. The deficits observed in the present study are subtle and unlikely to be apparent to individuals in the performance of everyday tasks. However, the present findings suggest that adults with type 1 diabetes who have developed IAH may have a reduced cognitive reserve compared with those with NAH, which may render them more susceptible to experiencing subsequent cognitive decline and associated educational and occupational challenges.

These findings suggest that frequent exposure to SH, as experienced by people with IAH, may underlie the observed cognitive impairments. However, causation cannot be determined from cross-sectional data. The lack of correlation between the frequency of SH episodes and cognitive test results may indicate that the observed association between IAH and cognitive deficits did not result from exposure to SH; instead, it might be explained by inaccurate recall of SH episodes, since it is known that retrospective estimation of hypoglycaemia is vulnerable to recall bias [40]. An association was found between the number of invalid tests and the number of SH episodes since diabetes onset in participants with IAH, which supports the hypothesis that recurrent SH may promote cognitive impairment.

The participants’ premorbid cognitive function was not assessed, and it is therefore not possible to establish whether (1) the cognitive impairment associated with IAH had resulted from recurrent exposure to SH, (2) premorbid cognitive impairment per se predisposed the individual to develop IAH, or (3) another common predisposing factor led to the simultaneous development of IAH and cognitive impairment. Since the IAH group did not exhibit impairment across all cognitive domains, but had significant impairments in tests of learning and memory that are associated with brain regions vulnerable to neuroglycopenia [11, 19, 20], the present results support a role for recurrent SH in the pathogenesis of IAH [9].

The awareness status of a few of the participants had changed between the cross-sectional study of 2011 and the present study. This is consistent with the dynamic nature of the IAH syndrome: awareness status may fluctuate and may even be restored by avoidance of hypoglycaemia [41]. When excluding the five participants whose hypoglycaemia awareness status had changed, group differences became more evident, thus demonstrating that persistent IAH status was most negatively associated with cognitive deficiency. Participants with IAH tended to have experienced more SH overall compared with participants with NAH and recorded more asymptomatic hypoglycaemia during the month preceding the study, consistent with the recognised characteristics of the IAH syndrome.

The strengths of the present study include the application of two validated methods to determine hypoglycaemia awareness status [42] and the use of an extensive battery of validated cognitive tests [25, 31, 35]. In addition, the use of strict criteria for inclusion in the statistical analyses excluded participants with IAH with the greatest performance impairments: only participants with IAH with the best cognitive function were compared with participants with NAH. Thus, the observed group differences in cognition between participants with IAH and NAH probably represented the minimum difference. The similar demographic and disorder-specific characteristics in the IAH and NAH groups, as well as in those participants who declined participation, are further strengths of this study. In the Norwegian Diabetes Registry [43], the average age, diabetes duration and HbA_1c_ level in people with type 1 diabetes was 41.8 years, 20.8 years and 8.0%, respectively, i.e. quite similar to the measures in the present study, which supports the generalisability of the present findings. Furthermore, the prevalence of microvascular complications and the level of educational attainment were similar in the IAH and NAH groups, and are therefore unlikely to have confounded the results.

The limitations of the study include the lack of measurement of participants’ premorbid cognitive function and the relatively modest sample size. While these may contribute to selection bias, there is no reason to believe that those eligible candidates who declined participation in the study had higher or lower cognitive abilities than people with type 1 diabetes in general. In addition, participants with NAH were chosen at random to reduce selection bias. Moreover, pre-test power analyses indicated that the proposed number of participants would be sufficient to yield clinically significant results.

It could be argued that participants should have been assessed using a continuous glucose monitoring system before commencing the study to identify asymptomatic biochemical hypoglycaemia that may influence cognitive function. Although cognition is impaired during hypoglycaemia and may remain abnormal for 40–75 min after hypoglycaemia has been treated [34], people with IAH have been shown to be less affected by hypoglycaemia compared with people with NAH and to recover more quickly [34]. As cognitive function is less affected by hypoglycaemia in people with IAH than those with NAH [34], any unrecognised biochemical hypoglycaemia in participants before the study would have been more likely to result in poorer performance in those with NAH, and would therefore not explain the present findings. Hyperglycaemia has also been found to impair cognitive function [44, 45], but an upper limit for the plasma glucose level was not specified before cognitive testing commenced. However, plasma glucose levels before and after testing were similar in the IAH and NAH groups (Table 1), with no association being found between elevated glucose levels and poorer cognitive performance.

As participants were not observed during cognitive testing, it is possible that they could have used aids when self-administering the cognitive tests, although they were instructed not to. The design of most tests made them impervious to attempts at cheating and the test platform did not allow individuals to redo tests. Based on the time stamps of keyboard strokes and the duration of each test session, it is very unlikely that any of the participants used aids while performing the tests.

The results of the present study are of considerable relevance to people with type 1 diabetes. The modest cognitive impairment observed in people with IAH may contribute to their increased risk of developing severe hypoglycaemia, and emphasises the necessity to reinforce structured education by using psychotherapeutic and behavioural therapies, and utilising diabetes technologies to avoid SH [46]. It has been suggested that impaired cognition may underlie the resistance shown by some people with IAH to co-operate in interventions to restore awareness of hypoglycaemia [47]. The present observations underline the value of including cognitive tests in intervention programmes to evaluate whether impaired cognitive ability may affect adherence to treatment and outcomes.

## Conclusion

The present study has demonstrated significant impairments in learning, memory, pattern separation and aspects of executive function (specifically, planning ability) in adults with type 1 diabetes who have IAH. These findings suggest that recurrent SH may have a role in promoting cognitive deficits in people with impaired hypoglycaemia awareness.

## Electronic supplementary material

Below is the link to the electronic supplementary material.

ESM (PDF 216 kb)

## Abbreviations

EDIC: Epidemiology of Diabetes Interventions and Complications
IAH: Impaired awareness of hypoglycaemia
IQR: Interquartile range
NAH: Normal awareness of hypoglycaemia
SH: Severe hypoglycaemia
SMBG: Self-monitoring of blood glucose
