# Supplementary material for: Cognitive deficits associated with impaired awareness of hypoglycaemia in type 1 diabetes
Source: Diabetologia. 2017 Mar 9;60(6):971–9. doi: 10.1007/s00125-017-4233-3 (PMC5423963; doi:10.1007/s00125-017-4233-3)
Supplement: Supplementary file 1 — (PDF 216 kb) [file 125_2017_4233_MOESM1_ESM.pdf]

Electronic supplementary material (ESM) Methods

Detailed information on cognitive tests employed

The six tests included in the cognitive test battery are described below. They were chosen based on their ability to assess distinct cognitive functions.

Verbal memory test: The participant is presented with a target list of 16 words read aloud one at a time. The words were chosen from four different semantic categories. Thereafter, the participant is instructed to recall and type the words from this list. There are four learning trials, followed by recall trials, which are followed by one distraction learning and recall trial with a similarly structured word list, but with other words. The participant is then instructed to recall words from the original target list (post-distraction recall) and ~15 minutes after completing other non-verbal tests, the participant completes a delayed recall trial. Performance is scored as number of correctly recalled words in each trial.

Objects in grid (object-location memory test): The participant is instructed to memorise the locations of 18 coloured line drawings of various objects within a 6 x 6 grid in 90 seconds. All objects are subsequently moved to the bottom of the screen. The participant is instructed to drag and drop the objects into their original places in the grid as a measure of immediate spatial recall. Performance is scored as the number of correctly placed objects.

Digit span backwards (working memory test): The participant is instructed to memorise a series of digits presented consecutively for 2 seconds each on the screen. After all digits in a trial have been presented, the participant is instructed to type the digits into a textbox in backward order using the keyboard, and when finished, click a button on the screen to continue to the next trial. The test consists of 18 trials, in which the number of digits to remember increases by one digit for every second trial starting with two digits in the first trial

and ending with ten digits for the last trial. The test ends if a participant makes three consecutive erroneous responses. Performance is scored as the number of correct trials.

Tower test (assesses planning abilities, a part of executive functions): The participant is shown an image on the screen depicting the goal positions of three different coloured discs located on three pegs and instructed to move a set of discs around on three other pegs on the screen below this picture. The discs can only be moved by following certain rules. The first peg can only hold one disc, the second peg can hold two discs and the third peg can hold three discs at any time. Further, only the top disc of a peg can be moved. Failing to comply with these rules, results in an illegal move. The test consists of 15 trials in sets of three that requires a minimum of one, two, three, four or five moves in order to reach the goal position. The test terminates automatically after 20 moves in one trial to prevent loss of motivation. Performance is scored as total number of moves used to solve all trials and total number of illegal moves performed.

Coding (information processing speed test with an executive component): In this test the participant is presented with a stimuli key of numbers and geometrical symbols on the top of the screen. Below are rows containing a random presentation of the geometrical symbols and empty cells. The first empty cell is highlighted and the participant is instructed to type in the corresponding number and repeat this process as fast and accurately as possible in 90 seconds. Performance is scored as the number of correct responses minus the number of erroneous responses.

Pattern separation (pattern separation abilities): In this test, the participant is presented with a series of images. For each image the participant is instructed to indicate whether the current image is identical to a previously presented image, similar to a previously presented image with a detail changed, or has never previously been presented in the image series. The test

consists of 108 trials (images), with 32 first presentations, 16 same presentations (identical), 16 similar (lure) presentations, and 44 unrelated presentations (foils). The score is calculated as the difference between the rates of “Similar” responses given to the lure items minus the rate of “Similar” responses given to the foils, which corrects for response biases.

The test order was fixed in the following way: Verbal memory test (distraction and post-distraction recall), Digit span backwards, Tower test, Verbal memory test (delayed recall), Objects in grid, Pattern separation, and Coding.

ESM Results

Performance on the cognitive tests after excluding five participants with altered hypoglycaemia awareness status

The IAH group scored significantly lower than the NAH group on the Verbal memory delayed recall (p=0.029, Cohen's d=−0.59 [95% CI −1.14, −0.1]), Objects in grid (p=0.009, Cohen's d=−0.66 [95% CI −1.16, −0.12]), Pattern separation (p=0.001, Cohen's d=−0.92 [95% CI −1.49, −0.37]), and Tower test log illegal moves (p=0.047, Cohen's d=0.61 [95% CI 0.02, 1.19]) tests. The IAH group also recalled fewer words on the Verbal memory test distraction list (p=0.011, Cohen's d=−0.68 [95% CI −1.19, −0.16]). No differences were found between the IAH and NAH group on the Verbal memory test post-distraction recall (p=0.228, Cohen's d=−0.31 [95% CI −0.86, 0.15]), Digit span backwards (p=0.471, Cohen's d=−0.19 [95% CI −.78, 0.27]), Tower test total moves (p=0.193, Cohen's d=0.39 [95% CI −0.19, 0.97]), or Coding (p=0.089, Cohen's d=−0.47 [95% CI −0.99, 0.08]) (ESM Table 1). The performance of the IAH and NAH groups across tests standardised with test norms are shown in ESM Fig. 1.

ESM Table 1 Cognitive test scores for the IAH and NAH groups after excluding five participants with altered hypoglycaemia awareness status

Cognitive test	IAH		NAH			
	n[a]	Mean ± SD	n[a]	Mean ± SD	*p*	Cohen's *d* (95% CI)
Verbal memory test[b]						
Distraction recall	27	6.4±2.4	35	8.3±3.1	0.011	−0.68 (−1.19, −0.16)
Post-distraction recall	27	12.3±2.5	35	13.2±2.6	0.228	−0.31 (−0.86, 0.15)
Delayed recall	27	12.4±2.1	32	13.7±2.1	0.029	−0.59 (−1.14, −0.1)
Objects in grid[b]	26	7.4±2.5	34	9.9±4.7	0.009	−0.66 (−1.16, −0.12)
Pattern separation[b]	23	0.18±0.20	33	0.35±0.17	0.001	−0.92 (−1.49, −0.37)
Digit span backwards[b]	25	9.2±2.7	33	9.9±2.8	0.471	−0.19 (−0.78, 0.27)
Coding[b]	23	29.6±8.1	34	34.4±11.6	0.089	−0.47 (−0.99, 0.08)
Tower test[c]						
Total moves	22	57.2±9.6	25	54.0±6.6	0.193	0.39 (−0.19, 0.97)
Log illegal moves	22	0.47±0.28	25	0.29±0.31	0.047	0.61 (0.02, 1.19)

[a]Column indicates number of valid responses
[b]Higher scores indicate better performance
[c]Higher scores indicate poorer performance

ESM Table 2 Characteristics of participants vs. subjects who declined participation in study ("Non-participants")

Parameter	Participants	Non-participants	p (95% CI)
	IAH (n=33)	IAH (n=23)	
HbA_{1c} level, %[a]	8.0±1.5	7.7±0.9	0.61 (−0.53, 0.98)
Age, years[a]	45.6±19.4	48.6±11.8	0.32 (−8.9, 2.9)
Diabetes duration, years	28.2±9.6	28.6±11.8	0.87 (−6.2, 5.3)
Sex (men: women), %[b]	42:58	61:30	0.17
SH preceding year, n (%)[b]			
0	15 (45.5)	7 (30.4)	0.50
1-2	10 (30.3)	8 (34.8)	
≥3	8 (24.2)	8 (34.8)	
	NAH (n=35)	NAH (n=8)	
HbA_{1c} level, %[a]	8.0±1.0	7.7±0.9	0.39 (−0.29, 0.73)
Age, years[a]	44.9±10.0	48.0±10.0	0.44 (−11.1, 4.9)
Diabetes duration, years	28.4±0.4	27.9±12.5	0.91 (−8.1, 9.1)
Sex (men: women), %[b]	40:60	25:75	0.43
SH preceding year, n (%)[b]			
0	27 (79.4)	5 (62.5)	0.28
1-2	5 (14.7)	3 (37.5)	
≥3	2 (5.9)	0	

Data are mean ± SD, unless otherwise stated
[a]Independent t test
[b]Chi square test

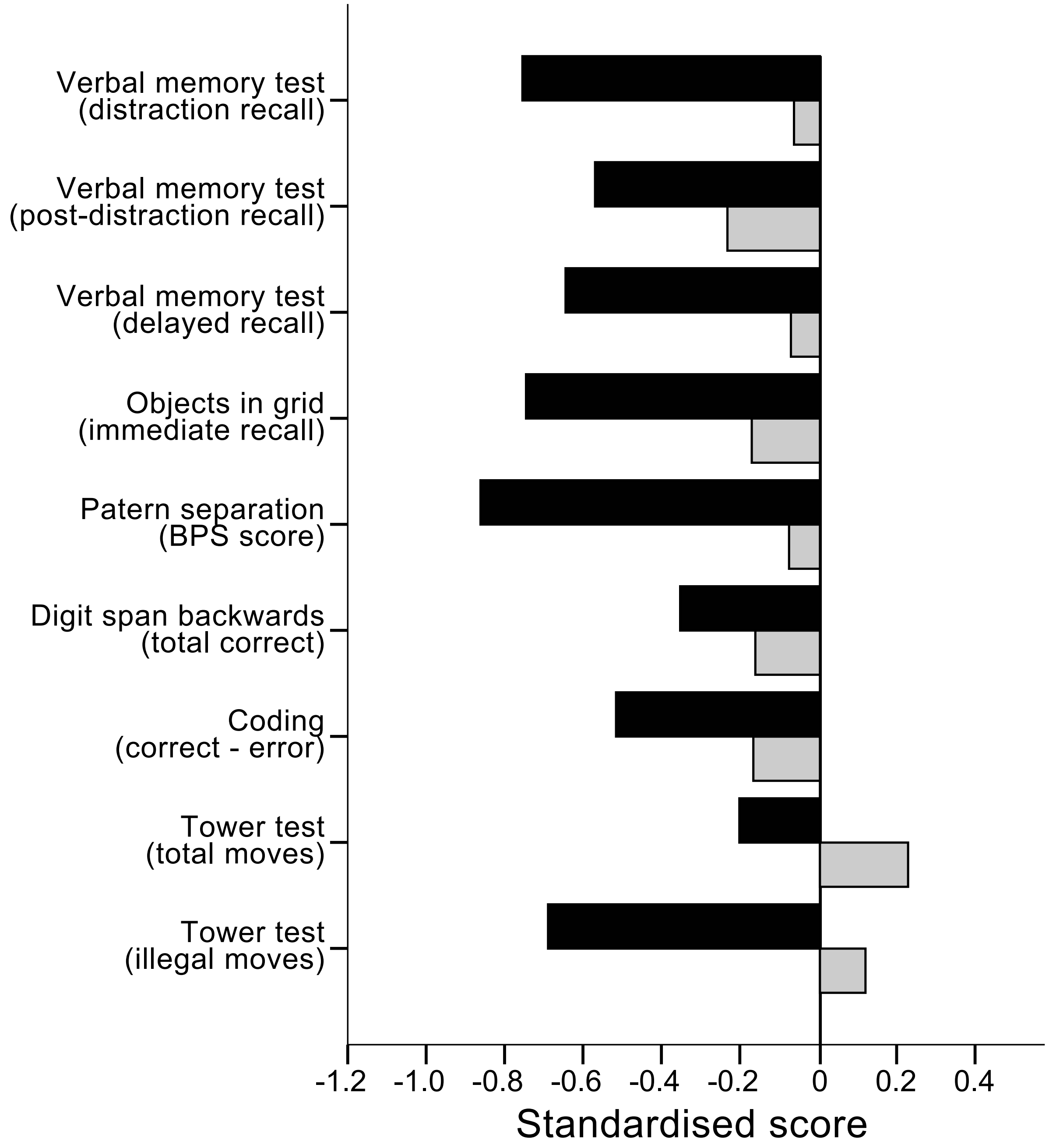

ESM Fig. 1 Scores for the nine cognitive measures (after excluding five participants with altered hypoglycaemia awareness status) were standardised against Memoro test norms for a non-diabetic population. Standardised scores for the Tower test were inverted such that a low value represents poorer performance. BPS, Behavioural pattern separation. Black bars, IAH. Grey bars, NAH.
